# Prognostic stratification of patients with AJCC 2018 pN1 disease in stage III oral squamous cell carcinoma

**DOI:** 10.1186/s40463-022-00573-x

**Published:** 2022-04-28

**Authors:** Ming-Hsien Tsai, Hui-Ching Chuang, Yu-Tsai Lin, Tai-Lin Huang, Fu-Min Fang, Hui Lu, Chih-Yen Chien

**Affiliations:** 1 Department of Otolaryngology, Kaohsiung Chang Gung Memorial Hospital and Chang Gung University College of Medicine, Kaohsiung, Taiwan; 2 Kaohsiung Chang Gung Head and Neck Oncology Group, Cancer Center, Kaohsiung Chang Gung Memorial Hospital, Kaohsiung, Taiwan; 3 College of Pharmacy and Health Care, Tajen University, Pingtung, Taiwan; 4 Department of Hematology and Oncology, Kaohsiung Chang Gung Memorial Hospital and Chang Gung University College of Medicine, Kaohsiung, Taiwan; 5 Department of Radiation Oncology, Kaohsiung Chang Gung Memorial Hospital, Chang Gung University College of Medicine, Kaohsiung, Taiwan; 6 Institute for Translational Research in Biomedicine, Kaohsiung Chang Gung Memorial Hospital, Kaohsiung, Taiwan

**Keywords:** Single nodal metastasis, N1, Stage III, Oral cancer, Prognosis

## Abstract

**Background:**

Oral cancer with pT1-3N1 without extracapsular extension of the lymph node is classified as stage III according to the eighth edition of the AJCC staging system. Outcomes of a subgroup of patients classified as having stage III oral cancer with single nodal metastasis are observed to be various clinically. Therefore, such clinical outcomes for subgroup analyses in this cohort are necessary.

**Methods:**

Patients with pT1-3N1 (based on the eighth edition of the AJCC staging system) oral cancer who underwent surgery between 2007 and 2016 were enrolled retrospectively for survival analyses.

**Results:**

A total of 105 patients—including 28 patients with pT1N1 disease and 77 patients with pT2-3N1 disease—participated in the study. Pathological T classification was the only statistically significant prognosticator according to univariate analysis. The patients with pT1N1 disease showed better 5-year overall survival (OS), disease specific survival (DSS), and disease free survival (DFS) than those with pT2-3N1 disease (pT1N1 vs pT2-3N1, OS: 96.4% vs 72.2%, *p* = 0.004; DSS: 96.4% vs 77.3%, *p* = 0.021; DFS: 84.6% vs 62.3%, *p* = 0.023). Besides, there was no potential clinicopathological confounder which is significant associated with different pathological T classifications in this unique cohort.

**Conclusions:**

Patients in the pT1N1 subgroup have significantly favorable prognosis than those with pT2-3N1 disease. Down-staging and reclassifying pT1N1 subgroup patients with oral cancer may be considered in tumor staging.

**Graphical Abstract:**

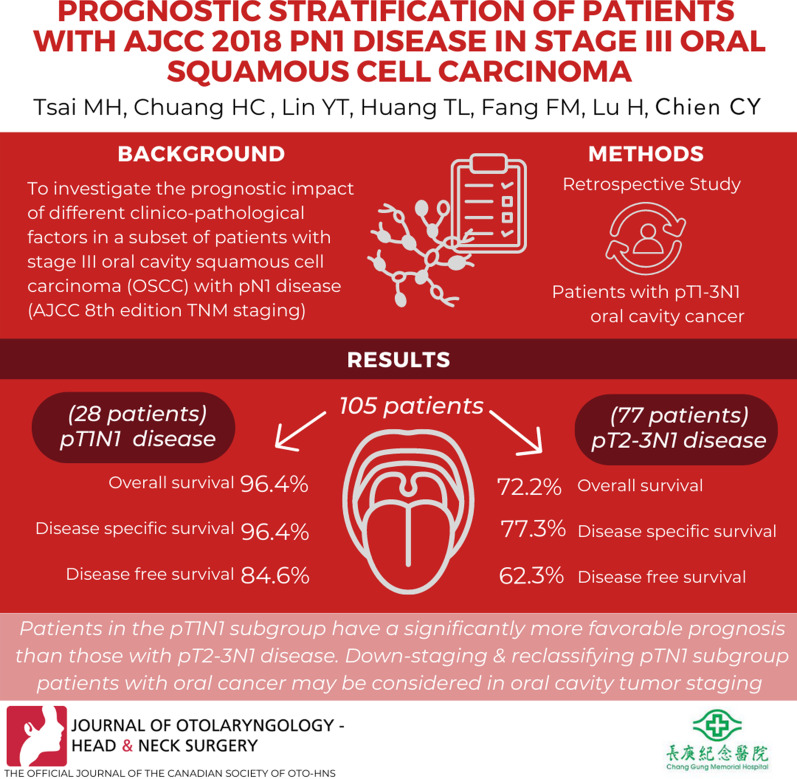

**Supplementary Information:**

The online version contains supplementary material available at 10.1186/s40463-022-00573-x.

## Background

Oral squamous cell carcinoma (OSCC) is a common malignant tumor in the head and neck region that accounts for 8.0% of all new cancers diagnosed and 6.3% of all cancer deaths annually in Taiwan [1]. Pathological nodal metastases are a well-known poor prognostic factor in patients with OSCC [2]. The presence of extranodal extension (ENE) was also recognized as a more adverse factor in patients with pathologically nodal metastases [3]. The newest American Joint Committee on Cancer (AJCC) staging system (eighth edition) introduced significant changes in terms of ENE classification to determine the N-classification of patients with OSCC [4]. The presence of ENE in previous pathologically confirmed pT1-3N1, stage III disease, would be upstaged to pT1-3N2a, stage IVa disease, respectively.

Given the upstaging of positive nodal status with ENE to stage IV disease in OSCC according to the AJCC eighth edition TNM staging system, the prognosis of stage III OSCC patients with pN1 disease is clinically fair. An improved prognostic stratification can help refine and tailor treatments at the individual level. Therefore, a detailed subgroup analysis of stage III OSCC patients with pN1 disease is necessary. We aimed to investigate the prognostic impact of different clinico-pathological factors in this subgroup of patients according to the AJCC eighth edition TNM staging system.

## Materials and methods

### Study population

Consecutive 1,476 treatment naive patients with OSCC were enrolled from the head and neck database, and they underwent upfront surgery with ipsilateral or bilateral neck dissections at Kaohsiung Chang Gung Memorial Hospital, Taiwan, between January 2007 and March 2016. The tumors were restaged according to the AJCC eighth edition TNM staging system, and the updated pN classification was as follows: pN0 (n = 1,018; 69%), pN1 (n = 127; 8.6%), pN2 (n = 139; 9.4%), and pN3b (n = 192; 13%). Patients who (a) did not receive curative intent surgery with neck dissection as an initial treatment strategy, (b) had any other previous cancer history, (c) had non-squamous cell carcinoma, and (d) had synchronous double cancer history were excluded from the study. In total, 105 patients were enrolled in our study cohort, including 28 patients with pT1N1 disease, 49 patients with pT2N1 disease, and 28 patients with pT3N1 disease. We also enrolled patients with pT2N0 disease from this cancer database during this period who served as the reference for outcome comparison.

### Neck dissections and postoperative adjuvant therapy

In general, classic radical or modified radical neck dissections (level I–IV) were performed in patients with clinically positive lymph nodes, whereas neck dissections (level I–III) were used in clinically node-negative patients. Patients were treated with bilateral neck dissections if the primary tumor reached or crossed the midline sagittal plane of the oral cavity. Treatment was mostly based on the American National Comprehensive Cancer Network (NCCN) guidelines. All the patients included in this study had detailed clinical and pathologic information, including adverse pathologic features, available for review. All the included patients completed the treatment strategy advised by the multidisciplinary team. The outcomes of interest investigated including overall survival (OS), disease-specific survival (DSS), and disease-free survival (DFS).

### Statistical analysis

Statistical analyses were performed using the Statistical Package for the Social Sciences software, version 25.0 (SPSS, IBM, Armonk, NY). The Kaplan–Meier method was utilized to estimate the probability of survival for each categorized factors, and the log-rank test was applied to examine the statistical significance of each factor to the survival outcomes. A two-tailed test with *p* value < 0.05 was considered statistically significant. This study was approved by the Medical Ethics and Human Clinical Trial Committees at Chang Gung Memorial Hospital. (Ethical Application Reference number:202101147B0).

## Results

A total of 105 patients with pN1 stage III OSCC were enrolled in this study. The clinical characteristics of the patients are summarized in Table 1. The median age of the patients was 54 years (range 32–77). The study sample included 91 (86.7%) male patients and 14 (13.3%) female patients. In tumor differentiation, most of the patients had moderately differentiated carcinoma (n = 62, 59%), followed by well-differentiated carcinoma (n = 38, 36.2%) and poorly differentiated carcinoma (n = 5, 4.8%). The median of the greatest tumor size was 22 mm (range 6–55), and the median of the depth of invasion (DOI) was 8 mm (range 1–19). Perineural invasion and lymphovascular invasion were reported in 32.4% and 21.9% of the patients, respectively. In total, 97 patients (92.4%) received ipsilateral neck dissection, while only 8 patients (7.6%) underwent bilateral neck dissection because of the cross midline of the tumor. Thirty-seven patients had clinical N0 disease and received neck dissections. Furthermore, 35 patients received adjuvant radiotherapy after surgery, 17 patients received adjuvant concurrent chemoradiotherapy, and 53 patients preferred not to receive any postoperative adjuvant therapy. Our study cohort included 28 patients (26.7%) with pT1N1 disease, 49 patients (46.6%) with pT2N1 disease, and 28 patients (26.7%) with pT3N1 disease. The patients were followed up for a median of 69.5 months in this cohort. Tumor recurrence was observed in 35 (33.3%) patients, including local, regional, locoregional recurrence and distant metastasis occurred in 11, 16, 4, and 4 patients, respectively.

**Table 1:** Patient characteristics (n = 105)

Characteristics	Value	%
Median age [range], yr	54 (32, 77)	
Median Follow up period [range], month	69.5 [6.2, 154.1]	
Sex		
Male	91	86.7
Female	14	13.3
Cancer location		
Tongue	41	39.0
Buccal	37	35.2
Other subsites	27	25.7
pT classification		
pT1	28	26.7
pT2	49	46.7
pT3	28	26.7
Histological grade		
WDSCC^†^	38	36.2
MDSCC^‡^	62	59
PDSCC^§^	5	4.8
Perineural invasion		
Positive	34	32.4
Negative	71	67.6
Lymphovascular invasion		
Positive	23	21.9
Negative	82	78.1
Surgical margin		
< 4 mm	23	21.9
≥ 4 mm	82	78.1
Median of depth of invasion [range], mm	8 [1, 19]	
Median value of positive node size [range], cm	1.3 [0.2, 3]	
Recurrence		
Yes	35	33.3
No	70	66.7

^†^WDSCC, well differentiated squamous cell carcinoma

^‡^MDSCC, moderately differentiated squamous cell carcinoma

^§^PDSCC, poorly differentiated squamous cell carcinoma

For pT classification, we found that patients in pT1N1 group had favorable outcomes than pT2N1 and pT3N1 groups (pT1N1 vs. pT2N1, OS: *p* = 0.009; DSS: *p* = 0.035; DFS: *p* = 0.039; pT1N1 vs pT3N1, OS: *p* = 0.001; DSS: *p* = 0.013; DFS: *p* = 0.023). However, pT2N1 group and pT3N1 group had very closed trend of Kaplan Meier survival curves (pT2N1 vs. pT3N1, OS, *p* = 0.252; DSS: *p* = 0.555; DFS: *p* = 0.717) (See Additional file 1: Table S1). Under this circumstance, we categorized our cohort into pT1N1 and pT2-3N1 as pT classification risk factor for further analysis.

**Fig. 1:**
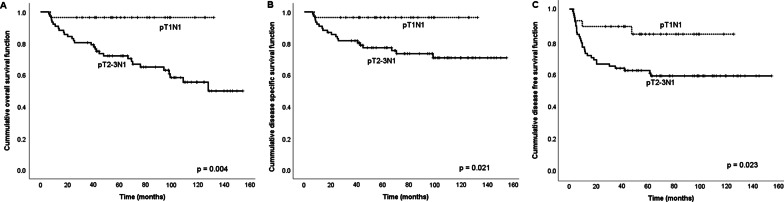
Kaplan–Meier survival curves according to pT classification. **A** Overall survival curves, **B** disease-specific survival curves, and **C** disease-free survival curves

The five-year survival rates of OS, DSS, and DFS were estimated for all clinico-pathological variables listed in Table 2. These results showed that pT1N1 disease was significantly associated with higher rates of five-year OS, DSS, and DFS compared with pT2-3N1 disease in univariate analysis (OS: *p* = 0.004, DSS: *p* = 0.021, and DFS: *p* = 0.023). The Kaplan–Meier curves of each strata (pT1N1 vs. pT2-3N1) was shown in Fig. 1. We also examined the relationships between the pT classification and other clinicopathological factors. The pT classification did not have significant association with age, histologic grade, PNI, LVI, surgical margin, positive nodal size and received adjuvant therapy or not (all *p* > 0.05) (See Additional file 2: Table S2).

**Table 2:** Univariate analysis of factors impacting survival (n = 105)

Variable	Number	5-year OS^†^	*p*	5-year DSS^‡^	*p*	5-year DFS^§^	*p*
		(%)		(%)		(%)	
Sex							
Male	91	77.2	0.780	80.8	0.233	66.6	0.310
Female	14	85.7		92.9		78.6	
Age							
< 54	50	85.7	0.140	85.7	0.134	71.9	0.281
≥ 54	55	71.8		79.3		65.0	
Smoking habit							
No	19	73.3	0.656	83.1	0.606	68.0	0.782
Yes	86	79.7		82.3		68.3	
Betelnut chewing							
No	22	71.0	0.603	85.2	0.511	72.4	0.473
Yes	83	80.3		81.5		67.1	
Alcohol drinking							
No	26	88.5	0.213	96.0	0.086	76.3	0.331
Yes	79	75.2		78.0		65.6	
pT classification							
pT1	28	96.4	0.004^*^	96.4	0.021^*^	84.6	0.023^*^
pT2-3	77	72.2		77.3		62.3	
Histological grade							
WDSCC^¶^	38	83.9	0.743	86.5	0.839	68.3	0.309
MDSCC^∥^	62	75.0		80.1		70.6	
PDSCC^#^	5	80.0		80.0		40.0	
Depth of invasion							
< 8 mm	52	84.1	0.121	88.5	0.256	74.6	0.351
≥ 8 mm	53	73.0		76.6		62.1	
Perineural invasion							
No	71	77.7	0.528	80.9	0.405	64.3	0.128
Yes	34	79.3		85.3		76.2	
Lymphovascular invasion							
No	82	78.7	0.953	81.2	0.857	66.6	0.621
Yes	23	77.7		86.7		73.9	
Surgical margin							
< 4 mm	23	71.2	0.279	71.2	0.320	60.9	0.364
≥ 4 mm	82	80.1		85.2		70.3	
Size of positive node							
< 1.3 cm	42	83.1	0.431	88.1	0.224	75.8	0.182
≥ 1.3 cm	63	75.1		78.5		63.3	
Adjuvant therapy							
No	53	72.5	0.801	78.1	0.239	63.5	0.270
Yes	52	84.3		86.5		73.0	

^†^OS, overall survival; ^‡^DSS, disease specific survival; ^§^DFS, disease free survival

^¶^WDSCC, well differentiated squamous cell carcinoma

^∥^MDSCC, moderately differentiated squamous cell carcinoma

^#^PDSCC, poorly differentiated squamous cell carcinoma

### Role of adjuvant therapy in subgroups of pN1 disease

In this cohort, no significant difference in survival was observed (all *p* > 0.05) between patients who received adjuvant therapy and those who did not in both pT1N1 and pT2-3N1 strata. In pT2-3N1 subgroup, the survival outcomes for the surgery-with-adjuvant-therapy patients and the surgery-alone patients had no significant difference (OS: 80.1% vs. 63.2%, *p* = 0.671, DSS: 82.9% vs. 70.6%, *p* = 0.182, and DFS: 68.2% vs. 55.6%, *p* = 0.175). Similar results were observed for pT1N1 stratum, the survival rates for surgery-with-adjuvant-therapy group vs. the surgery-alone group were, OS: 100% vs. 94.1%, *p* = 0.421; DSS: 100% vs. 94.1%, *p* = 0.421, and DFS: 90.9% vs. 80.2%, *p* = 0.56 (Table 3).

**Table 3:** Univariate Analysis of adjuvant therapy or not Impacting Survival (n = 105)

Variable	Number	5-year OS^†^	*p*	5-year DSS^‡^	*p*	5-year DFS^§^	*p*
		(%)		(%)		(%)	
pT1N1							
Without AT^¶^	17	94.1	0.421	94.1	0.421	80.2	0.560
With AT	11	100.0		100.0		90.9	
pT2-3N1							
Without AT	36	63.2	0.671	70.6	0.182	55.6	0.175
With AT	41	80.1		82.9		68.2	

^†^OS, overall survival; ^‡^DSS, disease specific survival; ^§^DFS, disease free survival; AT^¶^, adjuvant therapy

### Five-year OS, DSS, and DFS in OSCC patients with pT1N1 and pT2N0 disease

**Fig. 2:**
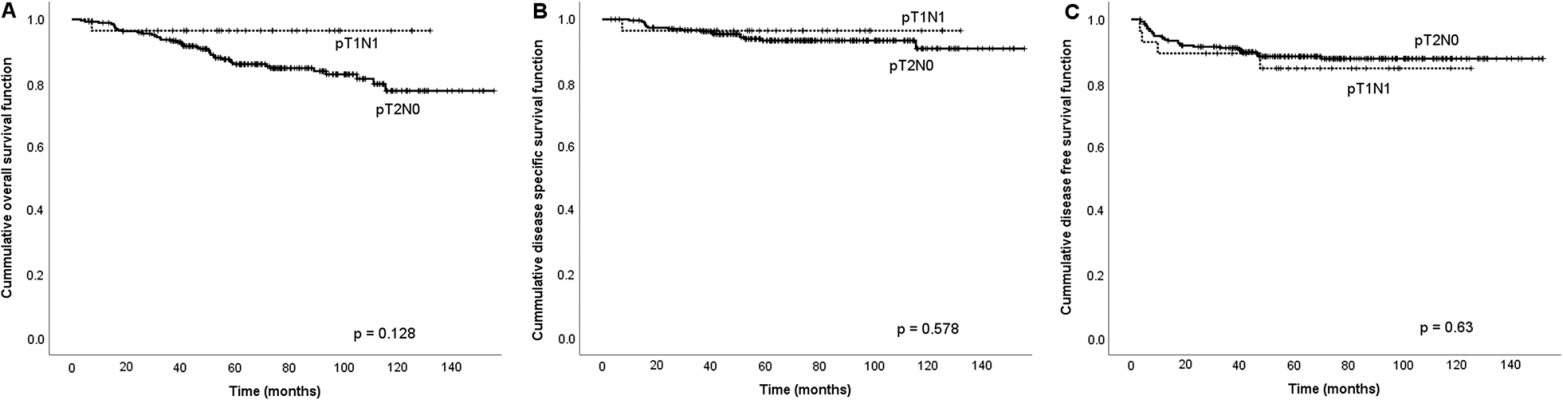
Kaplan–Meier survival curves of OSCC patients with pT1N1 disease and pT2N0 disease. **A** Overall survival curves, **B** disease-specific survival curves, and **C** disease-free survival curves

Given the significantly better survival of pT1N1 disease patients among those with stage III OSCC, another cohort of patients with pathologically confirmed T1N1 disease (n = 28) and T2N0 disease (stage II, n = 270) from our cancer database between January 2007 and March 2016 were enrolled for comparison in terms of survival rates. The outcomes showed no significant differences in terms of five-year OS, DSS, and DFS between pT1N1 (n = 28) and pT2N0 (n = 270) patients (OS: 96.4% vs. 84.8%, *p* = 0.128; DSS: 96.4% vs 93.7%, *p* = 0.578; DFS: 85.7% vs. 88.5%, *p* = 0.63). The Kaplan–Meier curves of OS, DSS, and DFS in patients with pT1N1 and pT2N0 disease (according to the AJCC eighth edition TNM staging system) were represented in Fig. 2.

## Discussion

The AJCC eighth edition TNM staging system recommended changes in the primary tumor and nodal staging of OSCC, including the DOI to the pT classification and ENE to the pN classification [4]. Although a significant improvement in precision in staging was observed, some limitations of the AJCC staging system were not addressed. Matos et al. [5] compared the AJCC seventh and eighth editions for OSCC in a cohort of 298 patients and found that patients upstaged by the DOI improved discrimination among pT1, pT2, and pT3 for DFS and OS; Garcia et al. [6] compared the pathological lymph node staging systems for multiple subsites of head and neck SCC, of which 270 patients had OSCC. They also found that patients upstaged by ENE had significantly worse cancer-specific survival than those who were not. However, the outcomes in the subgroup of stage III OSCC with pN1 disease (pT1N1, pT2N1, and pT3N1) varied clinically.

To the best of our knowledge, this study is the first to explore outcomes in pN1 with stage III OSCC among patients with different T classifications according to the AJCC eighth edition TNM staging system. A subgroup analysis of patients with stage III OSCC revealed that the clinical outcomes of pT1N1 disease showed lower HRs in OS, DSS, and DFS than the other groups (OS: *p* = 0.021, DSS: *p* = 0.049, and DFS: *p* = 0.028). We also found that prognosis of pT1N1 group and pT2N0 group didn’t have statistically significant difference. It provided the possibility of downstage for this unique subgroup patients, pT1N1, to stage II in the future staging system.

For OSCC patients with pN1 disease, the benefits of postoperative adjuvant therapy were still unclear. Chen et al. [7] analyzed 59 patients with pT1/pT2 with either pN0 or pN1 oral tongue squamous cell carcinoma, and they found improved five-year DSS in those who had undergone postoperative radiotherapy (PORT) compared with those had not after operation (81.2% vs. 53.0%; *p* = 0.03), but no difference in OS was observed (77.0% vs. 70.5%; *p* = 0.36). Shrime et al. [8] analyzed 1,539 patients in the Surveillance, Epidemiology, and End Results (SEER) database with only T1N1 and T2N1 OSCC, and they found that 78.6% of the patients had undergone PORT and that PORT was associated with improved five-year OS (54.2% vs. 41.4%; *p* < 0.001). Kao et al. [9] found no difference in five-year OS between pN1 OSCC patients who had and had not undergone PORT (38.7% vs. 36.0%; *p* = 0.23) using the SEER database. However, few studies have mentioned DOI and ENE status, which may alter staging according to the AJCC eighth edition staging system. In this cohort, OSCC patients with pN1 disease did not show significant alteration in survival related to postoperative adjuvant therapy. Interestingly, while we divided the study cohort into subgroups of pT1N1 and pT2-3N1 disease, patients with pT2-3N1 disease who had undergone adjuvant therapy had better oncologic outcomes than those who had not. No survival benefit of postoperative adjuvant therapy in pT1N1 disease was found.

Several limitations should be addressed in this study. First, this is a retrospective, single-institute study, and all the patients underwent surgical procedures at a single institution performed by different head and neck surgeons. Second, the case number in this study is limited. It may be the reason that none of the other adverse features such as DOI, PNI, LVI, margin status, and size of nodes were identified as significant prognosticator in this cohort. Besides, lack of multivariate analysis to adjust possible confounders was also another limitation of our study. Further multicenter studies are necessary to further confirm whether patients with pT1N1 disease have better clinical outcomes than those of other subgroups of stage III OSCC.

## Conclusions

Our data revealed that patients with pT1N1 disease have more favorable prognoses than pN2-3N1 patients among those with stage III OSCC. Down-staging patients with pT1N1 disease, along with reclassification in terms of tumor stage, may be considered in the future.

## Supplementary Information

**Additional file 1: Table S1**. Univariate analysis of different pT classification impacting survival.

**Additional file 2: Table S2**. Patient characteristics between pT1N1 and pT2-3N1 patients.

## Data Availability

The datasets used and/or analyzed during the current study are available from the corresponding author on reasonable request.

## Abbreviations

OSCC: Oral squamous cell carcinoma
ENE: Extranodal extension
AJCC: American Joint Committee on Cancer
NCCN: National Comprehensive Cancer Network
OS: Overall survival
DSS: Disease-specific survival
DFS: Disease-free survival
HR: Hazard ratio
CI: Confidence interval
PORT: Postoperative radiotherapy
SEER: Surveillance, Epidemiology, and End Results
