# Supplementary material for: Prognostic stratification of patients with AJCC 2018 pN1 disease in stage III oral squamous cell carcinoma
Source: J Otolaryngol Head Neck Surg. 2022 Apr 28;51:18. doi: 10.1186/s40463-022-00573-x (PMC9052608; doi:10.1186/s40463-022-00573-x)
Supplement: Supplementary file 1 — Additional file 1: Table S1. Univariate analysis of different pT classification impacting survival. [file 40463_2022_573_MOESM1_ESM.docx]

| Table S1. Univariate analysis of different pT classification impacting survival. | | | | | | | |
| --- | --- | --- | --- | --- | --- | --- | --- |
| Variable | | 5 yr OS^†^ (%) | *p* | 5 yr DSS^‡^ (%) | *p* | 5 yr DFS^§^ (%) | *p* |
| pT classification | pT1N1 | 96.4 | 0.009^*^ | 96.4 | 0.035^*^ | 84.6 | 0.039^*^ |
|  | pT2N1 | 74.6 |  | 80.7 |  | 65.3 |  |
| pT classification | pT1N1 | 96.4 | 0.001^*^ | 96.4 | 0.013^*^ | 84.6 | 0.023^*^ |
|  | pT3N1 | 67.7 |  | 71.4 |  | 56.9 |  |
| pT classification | pT2N1 | 74.6 | 0.252 | 80.7 | 0.555 | 65.3 | 0.717 |
|  | pT3N1 | 67.7 |  | 71.4 |  | 56.9 |  |
| Abbreviations: OS^†^, overall survival; DSS^‡^, disease specific survival; DFS^§^, disease free survival. | | | | | | | |
