# Supplementary material for: Prognostic stratification of patients with AJCC 2018 pN1 disease in stage III oral squamous cell carcinoma
Source: J Otolaryngol Head Neck Surg. 2022 Apr 28;51:18. doi: 10.1186/s40463-022-00573-x (PMC9052608; doi:10.1186/s40463-022-00573-x)
Supplement: Supplementary file 2 — Additional file 2: Table S2. Patient characteristics between pT1N1 and pT2-3N1 patients. [file 40463_2022_573_MOESM2_ESM.docx]

| Table S2. Patient characteristics between pT1N1 and pT2-3N1 patients. | | | | |
| --- | --- | --- | --- | --- |
| Variable | | pT subgroup | | p value |
|  |  | pT1N1 (n=28) | pT2-3N1 (n=77) |  |
| Age | < 54 | 12 | 38 | 0.556 |
|  | ≥ 54 | 16 | 39 |  |
| Sex | male | 26 | 65 | 0.344 |
|  | female | 2 | 12 |  |
| Smoking habit | no | 3 | 16 | 0.236 |
|  | yes | 25 | 61 |  |
| Betelnut chewing | no | 4 | 18 | 0.311 |
|  | yes | 24 | 59 |  |
| Alcohol drinking | no | 7 | 19 | 0.973 |
|  | yes | 21 | 58 |  |
| Histologic Grade | WDSCC^†^ | 6 | 32 | 0.053 |
|  | MDSCC^‡^ | 19 | 43 |  |
|  | PDSCC^§^ | 3 | 2 |  |
| PNI^¶^ | no | 23 | 48 | 0.055 |
|  | yes | 5 | 29 |  |
| LVI^∥^ | no | 22 | 60 | 0.943 |
|  | yes | 6 | 17 |  |
| Margin | < 4 mm | 8 | 15 | 0.319 |
|  | ≥ 4mm | 20 | 62 |  |
| Size of positive node | < 1.3 cm | 10 | 32 | 0.589 |
|  | ≥ 1.3 cm | 18 | 45 |  |
| Adjuvant therapy | none | 17 | 36 | 0.206 |
|  | yes | 11 | 41 |  |
| Abbreviations: WDSCC^†^, well differentiated squamous cell carcinoma; | | | | |
| MDSCC^‡^, moderately differentiated squamous cell carcinoma; | | | | |
| PDSCC^§^, poorly differentiated squamous cell carcinoma; | | | | |
| PNI^¶^, perineural invasion; LVI^∥^, lymphovascular invasion. | | | | |
